# Mechanistic Wound Healing of *Ficus trijuja* Leaf Extract and Its Lipid Nanocapsule Supported by Metabolomic Profiling and In Vivo Studies

**DOI:** 10.3390/ijms26030928

**Published:** 2025-01-23

**Authors:** Ingy M. Hashad, Shaza H. Aly, Dalia O. Saleh, Nesma M. E. Abo El-Nasr, Marwa E. Shabana, Fatma Sa’eed El-Tokhy, Heba A. S. El-Nashar, Usama R. Abdelmohsen, Nada M. Mostafa, Ahmed M. Mostafa

**Affiliations:** 1 Department of Biochemistry, Faculty of Pharmacy and Biotechnology, German University in Cairo, Cairo 11835, Egypt; 2 Department of Pharmacognosy, Faculty of Pharmacy, Badr University in Cairo (BUC), Cairo 11829, Egypt; 3 Department of Pharmacology, National Research Centre (NRC), Cairo 12622, Egypt; 4 Department of Pharmaceutics and Pharmaceutical Technology, Faculty of Pharmacy, Badr University in Cairo (BUC), Cairo 11829, Egypt; 5 Department of Pharmacognosy, Faculty of Pharmacy, Ain Shams University, Cairo 11566, Egypt; 6 Department of Pharmacognosy, Faculty of Pharmacy, Deraya University, New Minia 61111, Egypt; 7 Department of Pharmacognosy, Faculty of Pharmacy, Minia University, Minia 61519, Egypt; 8 Department of Biochemistry, Faculty of Pharmacy, Ain Shams University, Cairo 11566, Egypt

**Keywords:** *Ficus trijuja*, antioxidant, lipid nanocapsule, wound healing, flavonoids, terpenoids

## Abstract

This study explores the metabolic profile and wound-healing capabilities of *Ficus trijuja*, a species within the Moraceae family, for the first time. *Ficus* plants contain a variety of secondary metabolites such as flavonoids, triterpenoids, and alkaloids, known for their antioxidant, anti-inflammatory, and cytotoxic properties. Previous studies demonstrated the effectiveness of *Ficus* extracts in wound healing, with evidence of improved wound contraction, strength, and faster epithelization. This study shows the impact of *F. trijuja* extract in a gel base as well as when delivered through a lipid nanocapsules (LNCs) formula, on all phases of wound-healing in rats, by determining the expression levels of their key markers. The results indicated that treatment with *F. trijuja* extract accelerated wound healing, particularly when applied through lipid nanocapsulation, which shows comparable efficacy to standard treatments like MEBO^®^ with approximately 2.62% improvement in wound healing when compared to MEBO^®^ itself. Understanding these molecular markers offers insights for developing targeted therapies to optimize wound healing and prevent complication development. To unravel the phytochemical composition of *F. trijuja* extract, LC-HRESIMS analysis was implemented, which revealed 24 secondary metabolites belonging to different classes of flavonoids, terpenoids, and megastigmane. In conclusion, both *Ficus trijuja* gel and its nanoformulation have proven efficacy in wound healing in vivo and can be further investigated for potential clinical use.

## 1. Introduction

*Ficus trijuja* belongs to the genus *Ficus* in the Moraceae family, widely known as the mulberry family or the fig family. It is a flowering plant family with approximately 40 genera and 1000 species [1]. The genus *Ficus* is one of the important genera in the family Moraceae due to its high economic and nutritional values. It encompasses over 800 species, and previous studies revealed the isolation of many secondary metabolites that belong to various classes such as flavonoids, triterpenoids, alkaloids, and stilbenes [2,3,4,5]. Different species in the genus *Ficus* are reported for a plethora of biological activities including but not limited to antioxidant, anti-inflammatory, antimicrobial, antidiabetic, and cytotoxic activities [5,6,7,8,9]. Numerous studies have investigated the wound-healing properties of various *Ficus* species [10,11,12,13,14]. For instance, a recent study has shown that the extract of *F. carica* fruit-loaded amphiphilic polymeric scaffold can enhance the diabetic-affected wound [15]. The aqueous extract of *F. benghalensis* roots showed enhancement in the breaking strength and reduced the epithelization period along with elevation in would contraction percentage [16]. Another study revealed that the acetone extract of leaves of *F. amplissima* had potent wound-healing properties through antioxidant and anti-inflammatory effects that were attributed to the presence of high phenolic contents [16].

Many formulations and studies have investigated the role of herbal extracts to ensure their efficacy, availability, and safety in the management of the wound healing process [17,18,19]. Wound healing is a complex and dynamic process of body tissue repair involving several stages that require a detailed understanding for optimal patient care. These stages can be broadly categorized into four interrelated phases: hemostasis, inflammation, proliferation/migration, and maturation/remodeling [20,21,22].

Lipid nanocapsules (LNCs) are colloidal nanoparticles that emerged as an effective tool for the delivery of hydrophilic, lipophilic, and/or poorly water-soluble drugs as an alternative to liposomes, emulsions, and polymeric nanocarriers. The assembly of LNCs mainly comprises three components: the oily phase, non-ionic surfactants (hydrophilic and lipophilic), and aqueous phase [23]. The oily phase essentially consists of medium chain triglycerides (MCT) such as Labrafac™ PG, Miglyol^®^ 812, or Labrafil^®^ M1944CS. The hydrophilic surfactants such as Kolliphor^®^ HS15 (formerly Solutol^®^ HS15) are derived from polyethylene glycol (PEG) and are a mixture of free PEG 660 and PEG 660 hydroxystearate. Also, Kolliphor EL^®^ is a non-ionic solubilizer and emulsifier synthesized by reacting castor oil with ethylene oxide. Fatty acid esters of polyethylene glycol comprise the hydrophobic part of surfactant while free polyethylene glycols and ethoxylated glycerol represent the hydrophilic part. In addition, a small amount of lipophilic surfactant such as lecithin is commonly used. Lecithin was found to confer the hardness of the outer shell and increase the stability of lipid nanocapsules.

The process of wound healing relies on a complex interplay of cellular and molecular events, with various signaling molecules acting as pivotal markers in this process. Wound healing markers serve to provide information about the progression and success of the wound healing process. These markers consist of molecules such as growth factors, cytokines, and enzymes that direct and control the healing process. Examples of these important markers include Tumor Necrosis Factor-alpha (TNF-α), interleukin 1Beta (IL1β) Vascular Endothelial Growth Factor (VEGF), Platelet-Derived Endothelial Growth Factor (PDGF), Tissue Inhibitor of Metalloproteinases 2 (TIMP2), and Matrix Metalloproteinase 2 (MMP2) [24]. TNF-α, primarily known for its pro-inflammatory effects, is a double-edged sword in wound healing. In the early stages, it recruits immune cells to the wound site, aiding in debris removal. However, sustained TNF-α presence can lead to chronic inflammation, delaying the healing process. Thus, a balanced TNF-α response is crucial for optimal wound healing [25]. VEGF is the master orchestrator of angiogenesis, a vital process for wound healing ensuring the delivery of oxygen and nutrients to the healing tissue. VEGF also promotes endothelial cell migration, further accelerating wound closure [26]. PDGF plays a pivotal role in the proliferation of fibroblasts and smooth muscle cells, essential for tissue repair. It stimulates the production of collagen and fibronectin, contributing to wound closure and scar formation [27]. TIMP-2 and MMP-2 are part of a delicate balance responsible for ECM remodeling during wound healing. While MMP-2 degrades ECM components to facilitate cell migration, TIMP-2 counteracts MMP-2’s excessive activity, preventing aberrant tissue remodeling. The dynamic interaction between these molecules ensures precise tissue repair [28]. IL-1β is a pro-inflammatory cytokine that initiates the immune response at the wound site. It recruits leukocytes and activates endothelial cells, promoting inflammation. In controlled amounts, IL-1β aids in clearing pathogens and initiating tissue repair. However, excessive IL-1β can lead to chronic inflammation and impaired wound healing [29,30,31,32,33].

In summary, these wound healing markers all work in concert to ensure a well-coordinated healing process. Understanding these wound-healing markers at a molecular level provides a foundation for developing targeted therapies to enhance wound healing in clinical settings.

To the best of our knowledge, the wound-healing ability of the selected species, *Ficus trijuja*, has not been investigated before. That gives us the interest to explore its activity based on in vivo investigations as well as the histopathological effects. Moreover, the identification of its major secondary metabolites using LC-HRESIMS analysis demonstrates the biologically active compounds that may be involved in wound healing activity.

## 2. Results and Discussion

### 2.1. LC-HRESIMS Metabolomic Analysis

LC-HRESIMS profiling of *F. trijuja* leaves revealed the presence of various secondary metabolites as shown in (Table 1). The LC-HRESIMS-based chemical characterization of *F. trijuja* led to the dereplication of 24 major secondary metabolites in the extract (Figure S1). The dereplicated compounds (**1**–**24**) have been previously reported in different *Ficus* species [9,34,35,36,37,38]. Moreover, they were found to belong to flavonoids, terpenoids, and megastigmane derivatives. The total ion chromatogram of the total extract of *F. trijuja* (negative and positive ion mood) is represented in (Figures S2 and S3). The mass ion peak at *m*/*z* 379.08275 for the suggested formula C_21_H_16_O_7_ was identified as gancaonin F; 9-Hydroxy (**1**), which was formerly reported from roots of *Ficus hirta* [39]. The mass ion peak at *m*/*z* 193.07054 was assigned for the predicted molecular formula C_7_H_12_O_6_ and identified as Quinic acid (**2**) that was previously obtained from *Ficus sycomorus* [40]. Moreover, the mass ion peaks at *m*/*z* 335.09294, 166.08615, and 169.01432 corresponding to the suggested molecular formulas C_20_H_16_O_5_, C_9_H_11_NO_2_, and C_7_H_6_O_5_, respectively, were identified as carpachromene (**3**), phenylalanine (**4**), and gallic acid (**5**), which was previously isolated from *Ficus* sp. [41,42,43]. Additionally, the mass ion peaks at *m*/*z* 327.10715 and 469.33085 corresponding to the molecular formula C_15_H_18_O_8_ and C_30_H_44_O_4_ identified foveospirolide (**6**) and foveolide B (**16**) as sesquiterpenoid derivatives, respectively [44]. Furthermore, the mass ion peak at 225.1484 and 507.27947, in accordance with the molecular formulas C_13_H_20_O_3_ and C_24_H_42_O_11_, were dereplicated as megastigmane derivatives 9,10-dihydroxy-4,7- megastigmadien-3- one (**8**) and ficalloside (**9**), which were previously obtained from *F.pumila* and *F. callosa* [45]. Similarly, the mass ion peaks at *m*/*z* 539.21185, 595.16502, 611.15993, and 511.18053, for the predicted molecular formulas C_26_H_34_O_12_, C_27_H_30_O_15_, C_27_H_30_O_16_, and C_24_H_30_O_12_ were dereplicated as 3,3′,4,4′,5,5′,7-heptahydroxyflavan; 3′,4′,5,5′,7-Penta-Me ether, 3-O-α-L-rhamnopyranoside (**10**), two flavonoids and leucoanthocyanidin; sorbifolin-6-O-[*α*-L-arabinopyranosyl-(1→2)-*β*-D-glucopyranoside] (**11**), rutin (**12**), and 5, 7, 3 trimethoxy leucodelphinidin 3-O-*α*-L-rhamnoside (**13**), which were formerly characterized from different *Ficus* sp. [9,34,35,36]. Moreover, the molecular ion mass peaks at *m*/*z* 473.36217, 457.36732, 457.36733, 459.38296, 427.39316, 443.3881, and 441.37248 for the predicted molecular formulas C_30_H_48_O_4_, C_30_H_48_O_3_, C_30_H_48_O_3_, C_30_H_50_O_3_, C_30_H_50_O, C_30_H_50_O_2_, and C_30_H_48_O_2_ gave hits of the triterpenoids, 29,30-dinor-3*β*-acetoxy-18,19-dioxo-18,19-secolupane (**17**), 19,20-secoursane-3,19,20-trione (**18**), 3*α*-hydroxyisohop-22(29)-en-24-oic acid (**19**), 3*β*-hydroxy-11*α*-hydroperoxy-12-ursene (**21**), rhoiptelenol (**22**), 20*α*,21*α*-epoxytaraxastan-3*β*-ol (**23**), and 29(20→19) abeolupane-3,20-dione (**24**), respectively, which were previously isolated from different *Ficus* sp. [46,47,48,49,50].

LC-HRMS profiling is valuable for exploring complex samples and identifying various compounds present in the plant extract; quantitative LC/MS analysis is better suited for the precise quantification of known compounds with high sensitivity, accuracy, and reproducibility. The choice between the two approaches depends on the specific analytical goals and requirements of the study. As in our study, we make a tentative identification of the secondary metabolites by conducting LC-HRMS profiling of the *F. trijuja* extract, which is essential for unraveling the chemical complexity of plant extract and identifying its bioactive compounds.

### 2.2. Preparation and Characterization of F. trijuja Extract LNCs

**Figure 1:**
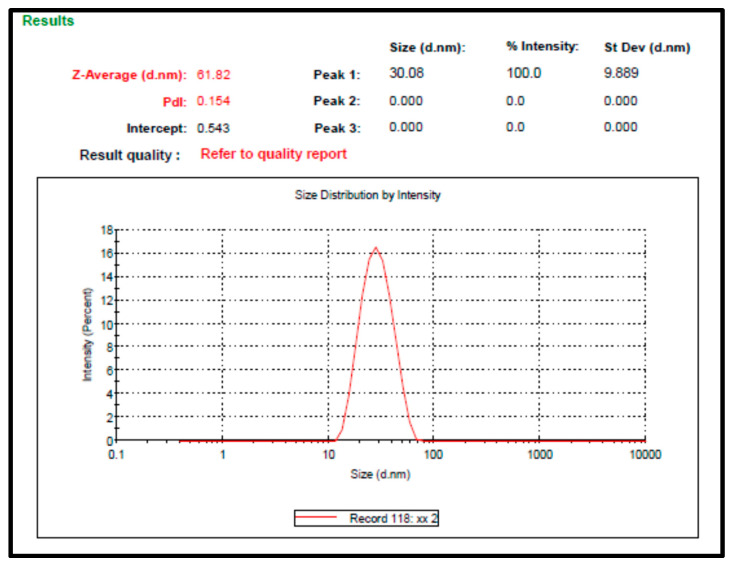
Particle size distribution of *F. trijuja* extract LNCs.

LNCs were successfully prepared and displayed a small particle size with low polydispersity index values (Figure 1), indicating the homogeneity of the prepared nanocarriers.

The Zeta potential of the LNCs loaded with herbal extract revealed that they are negatively charged (−27.3 mV ± 2.6). This charge could be related to the phospholipids incorporated in the outer shell along with the PEGylated surfactant. The negatively charged surfaces result in static repulsion between particles, reducing their tendency to aggregate.

The colloidal particles of LNCs are stabilized using the macrogols (high MWT surfactants) that employ steric hindrance between the suspended nanoparticles, thus preventing them from aggregation The excellent stability of LNCs is originated from their low PDI values (0.154), steric hindrance of hydrophilic surfactants, and negatively charged surface [23,54] (Figure 1).

### 2.3. Effect of F. trijuja Extract LNCs on the Promotion of Wound Healing

The impact of nanocarriers on improving the permeation of drugs through deeper skin layers has been thoroughly investigated. This impact could be obviously perceived by the higher wound healing effect of the loaded LNCs as proven by the in vivo study in this research. This result confirms the significant advancement in LNCs in wound healing applications, offering unique benefits compared to other nano-delivery systems such as solid lipid nanoparticles (SLNs), liposomes, and hydrogels. The small droplet size of LNCs allows for better interaction with microbial cell membranes. This leads to the effective disruption of bacterial cells, thereby reducing infection rates in wounds. Also, the high loading capacity of both hydrophilic and lipophilic drugs improves the overall therapeutic efficacy. This is particularly beneficial for delivering herbal extracts that require the application of higher concentrations at the wound site. Nevertheless, LNCs provide controlled drug release profiles, preventing the burst effect often seen with SLNs and ensuring that therapeutic agents are delivered steadily over time [55]. Their thermodynamic stability prevents phase separation and degradation, maintaining drug efficacy throughout the treatment period, unlike liposomes, which could lose their structural integrity over time [56,57]. Owing to their small particle size and lipid composition, LNCs can penetrate deeper into the skin layers compared to other systems like SLNs or liposomes. This enhanced permeation improves bioavailability and accelerates the healing process [58]. Interestingly, LNCs have been shown to enhance macrophage activation, which plays a vital role in the inflammatory phase of wound healing. This can lead to improved wound closure and tissue regeneration [55].

Our strategy to design this nanoformulation aimed to fulfill two main goals. The first of which is to prepare nanocarriers with small particle sizes that could entrap *F. trijuja* extract and greatly enhance its permeation via intercellular spaces and follicular pathways [59]. Therefore, LNCs were prepared due to their well-known small particle size range and low polydispersity index. The second goal is to select the components of the formulation so that they can act as penetration enhancers [60,61], as terpenes interact with the skin by replacing the hydrogen bonds between ceramides in the stratum corneum with the formation of new bonds between a hydrogen donor or acceptor group in terpenes and ceramides. This could lead to the formation of microcavities and an increase in the free volume for drug diffusion [62].

The second goal is to select the components of the formulation so that they boost wound healing by themselves in order to maximize the pharmacological effect of the loaded nanoformulation and achieve better clinical outcomes at lower concentrations of the active constituents. Consequently, Eucalyptus oil was selected as a carrier oily vehicle due to its known effect to promote wound healing through the promotion of neovascularization, the anti-inflammatory effect, and increased collagen production (Alam et al., 2018; Amer and Kadhim, 2018). Nevertheless, Eucalyptus oil is an essential oil with a high content of terpenes that act as penetration enhancers (Alam et al., 2018; Woollard et al., 2007). Terpenes interact with the skin by replacing the hydrogen bonds between ceramides in SC with the formation of new bonds between hydrogen donor or acceptor groups in terpenes and ceramides. This could lead to the formation of microcavities and to an increase in the free volume for drug diffusion (El-Tokhy et al., 2021).

Alam, P., Shakeel, F., Anwer, M. K., Foudah, A. I., and Alqarni, M. H. (2018). Wound Healing Study of Eucalyptus Essential Oil Containing Nanoemulsion in Rat Model. Journal of Oleo Science, 67(8), 957–968. https://doi.org/10.5650/jos.ess18005

Amer, H., and Kadhim, E. F. (2018). Laser Doppler Evaluation to the Effect of Local Application of Eucalyptus Oil on Wound Healing: Histological Study on Male Rats. International Journal of Medical Research and Health Sciences, 7(12), 114–120. www.ijmrhs.com

El-Tokhy, F. S., Abdel-Mottaleb, M. M. A., El-Ghany, E. A., and Geneidi, A. S. (2021). Design of long-acting invasomal nanovesicles for improved transdermal permeation and bioavailability of asenapine maleate for the chronic treatment of schizophrenia. International Journal of Pharmaceutics, 608. https://doi.org/10.1016/j.ijpharm.2021.121080

The current work introduces a new tool for the acceleration of wound healing as a novel herbal extract and its inclusion into nanocarriers, which could be thoroughly investigated in terms of mechanisms and kinetics in future studies.

### 2.4. Morphometric Evaluation of the Rat Skin After Treatment with F. trijuja Extract

**Figure 2:**
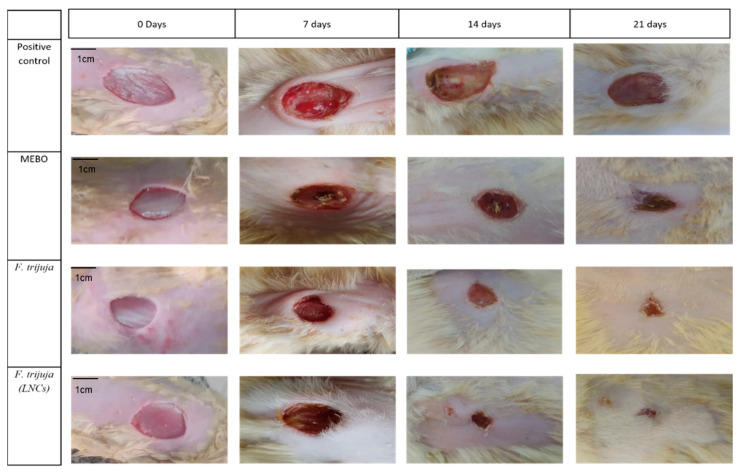
Morphometric evaluation of the rat skin after treatment with *F. trijuja* extract.

The healing process was assessed by observing lesions morphologically. At the end of each period (0, 7, 14, and 21 days), animals in each group were photographed for macroscopic evaluation (Figure 2).

**Figure 3:**
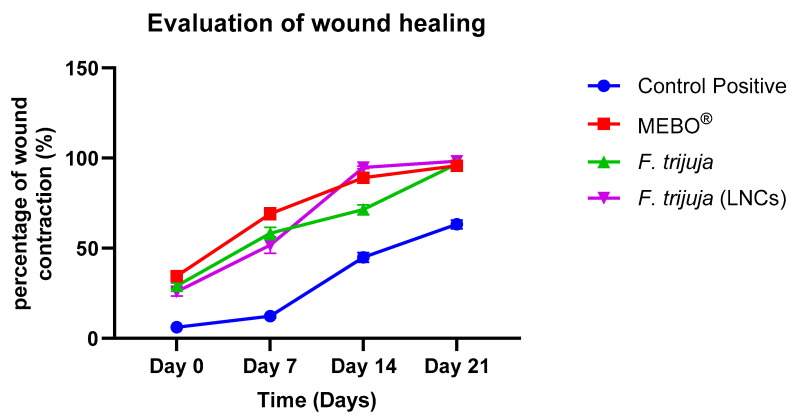
Effect of *F. trijuja* (in gel formulation) and *F. trijuja* (LNCs) treatment on the percentage of wound healing (wound contraction) on excision-induced wounds in rats.

The assessment of wound healing was evaluated by measuring the percentage reduction in wound size at 0, 7, 14, and 21 days after the start of the experiment. It was noticed that the wound healing rates increased with time in all groups. *F. trijuja* (LNCs) showed a significant increment in wound healing activity as compared to the control positive group (Table 2 and Figure 3).

The percentage improvement in wound healing compared to MEBO at 21 days was calculated using the formula:

(Healing with Treatment − Healing in Positive Control)/Healing in Positive Control × 100%

Substituting the values, (98.28 − 95.77)/95.77 × 100% ≈ 2.62%. This indicates that the addition of *F. trijuja* through LNCs improves wound healing by approximately 2.62% compared to the positive control (MEBO^®^).

### 2.5. Effect of F. trijuja Extract Treatment on the mRNA Expression of TNF-α and the Protein Expression of IL-1β in Wounded Rats

**Figure 4:**
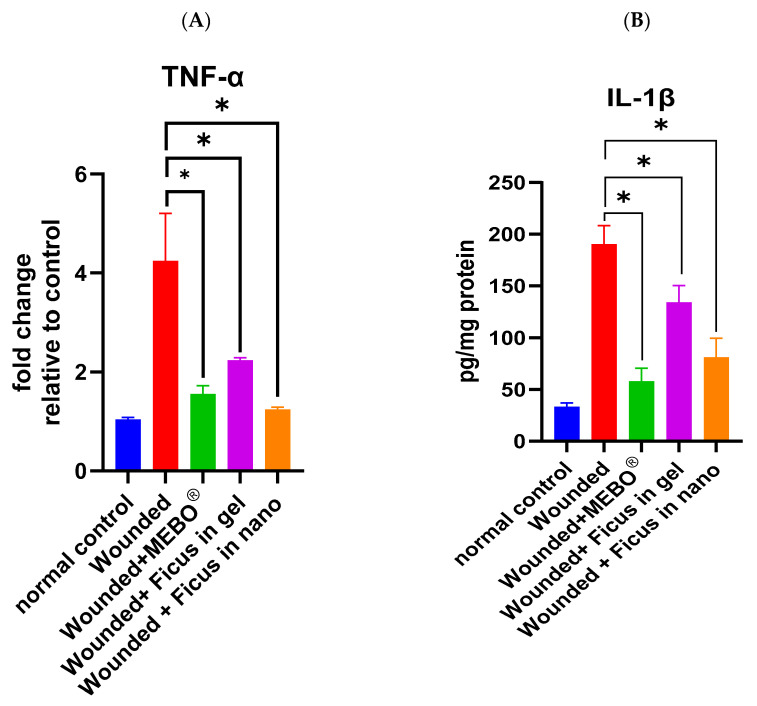
Effect of *F. trijuja* extract treatment on TNF-α (**A**) and IL-1β (**B**) levels in wounded rats. A total of n = 5 per group was used for all experimental groups to ensure statistical power and reproducibility. Values are expressed as Mean ± SD (n = 5) and analyzed by one-way ANOVA followed by Tukey’s post hoc multiple comparison test. * *p* < 0.05 as compared to the wounded positive control group.

In Figure 4A,B, the mRNA expression of TNF-α and the protein expression of IL-1β is depicted. Analysis of mRNA expression of TNF-α and the protein expression of IL-1β in wounded samples on day 21 post-injury showed a significant down-regulation of these inflammatory markers in wounds treated with *F. trijuja* extract (both in gel as well as in nano-formulation) and with MEBO^®^ compared to untreated wounds (*p* < 0.05). Notably, wounds treated with *F. trijuja* extract in nano-formulation exhibited a similar reduction in these inflammatory markers (TNF-α and IL-1β) compared to the MEBO^®^-treated group. The expression of TNF-α and IL-1β in wounds treated with *F. trijuja* extract was significantly lower even in the group treated with MEBO^®^ and nano-formulated *F. trijuja* extract than in the group treated with a gel-formulated *F. trijuja* extract, which exhibited that nano-formulations showed significantly higher efficiency in the treatment of wounds than gel-formulation. Pro-inflammatory cytokines like IL-1β and TNF-α play a crucial role in recruiting neutrophils to the wound site and inducing matrix metalloproteinase (MMP) production in inflammatory and fibroblast cells. Both TNF-α and IL-1β are primarily known for their pro-inflammatory effects; TNF-α serves as a dual-purpose factor in wound healing. Initially, it plays a beneficial role by attracting immune cells to facilitate debris removal at the wound site. However, the prolonged presence of TNF-α can result in persistent inflammation, consequently impeding the timely progression of the healing process. Thus, a balanced TNF-α response is crucial for optimal wound healing [25]; IL-1β functions by recruiting leukocytes and activating endothelial cells, thereby activating inflammation. In regulated quantities, IL-1β contributes to pathogen clearance and initiates the process of wound healing. However, the overexpression of IL-1β can result in chronic inflammation and impaired wound healing [29]. TNF-α stimulates NF-κB, which, in turn, promotes the expression of various pro-inflammatory cytokines and proteases such as MMPs [63]. Therefore, the suppression of inflammatory cytokines (TNF-α and IL-1β) by *F. trijuja* extract can inhibit prolonged inflammation and promote wound repair. These results collectively suggest that *F. trijuja* extract may expedite the transition from an inflammatory to an anti-inflammatory response.

### 2.6. Effect of F. trijuja Extract Treatment on the mRNA Gene Expression Levels of VEGF in Wounded Rats

**Figure 5:**
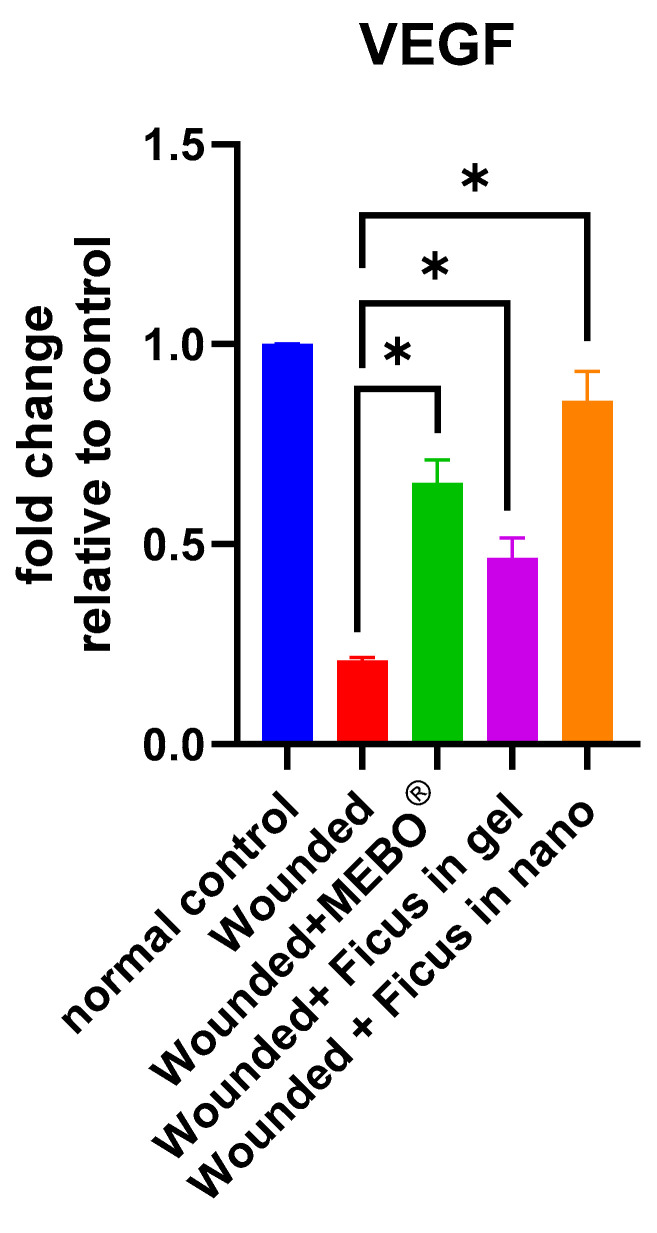
Effect of *F. trijuja* extract treatment on VEGF mRNA expression levels in wounded rats. A total of n = 5 per group was used for all experimental groups to ensure statistical power and reproducibility. Values are expressed as Mean ± SD (n = 5) and analyzed by one-way ANOVA followed by Tukey’s post hoc multiple comparison test. * *p* < 0.05 as compared to the wounded positive control group.

Figure 5 illustrates the mRNA gene expression levels of VEGF in response to excisional wound therapy, which involves the application of *F. trijuja* extract (both in gel form and as a nano-formulation) and MEBO^®^. In the skin tissues of wounds treated with *F. trijuja* extract for 21 days, a significant increase in VEGF gene expression was observed when compared to untreated wounds (*p* < 0.05). Notably, wounds treated with *F. trijuja* extract in the nano-formulation exhibited a similar increase in the expression of this marker when compared to the group treated with MEBO^®^. It is important to emphasize that the intricate interaction between cells and various growth factors is critical in the wound-healing process. During the process of wound healing, a diverse range of cell types, such as macrophages and fibroblasts, produce VEGF. This VEGF plays a pivotal role in numerous phases of the wound healing process. In the initial phases of healing, VEGF encourages vascular permeability and the expression of adhesion molecules, facilitating the recruitment of inflammatory cells. The levels of VEGF can affect the speed at which the wound closes and re-epithelializes, undergoes angiogenesis, forms granulation tissue, and determines the ultimate strength of the healed wound during the proliferative phase. Furthermore, VEGF can encourage the development of scar tissue as the remodeling phase takes place [26,64].

### 2.7. Effect of F. trijuja Extract Treatment on the mRNA Gene Expression Levels of PDGF in Wounded Rats

**Figure 6:**
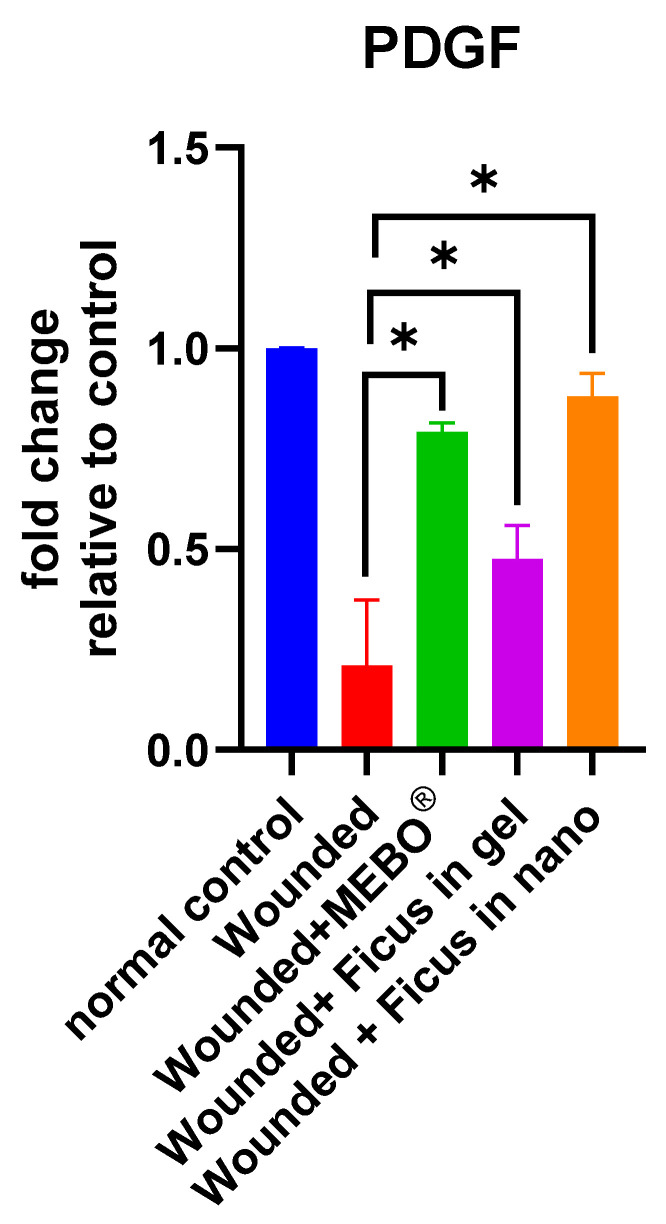
Effect of *F. trijuja* extract treatment on PDGF mRNA expression levels in wounded rats. A total of n = 5 per group was used for all experimental groups to ensure statistical power and reproducibility. Values are expressed as Mean ± SD (n = 5) and analyzed by one-way ANOVA followed by Tukey’s post hoc multiple comparison test. * *p* < 0.05 as compared to the wounded positive control group.

The data presented in Figure 6 illustrate the mRNA gene expression levels of PDGF during excisional wound therapy. This therapy involves the application of *F. trijuja* extract, both in gel and nano-formulation, along with MEBO^®^. After 21 days of treatment with *F. trijuja* extract, a significant rise in PDGF gene expression was observed in the skin tissues of the wounds compared to untreated wounds (*p* < 0.05). Importantly, wounds treated with *F. trijuja* extract in the nano-formulation exhibited a similar increase in the expression of this marker, comparable to the rise seen in the group treated with MEBO^®^. PDGF is a protein that plays a crucial role in the various stages of wound healing. PDGF is produced by platelets and various other cell types, including macrophages, endothelial cells, and smooth muscle cells. It contributes to the healing process through its effects on cell migration, proliferation, and the formation of blood vessels and connective tissue. During the initial inflammatory phase of wound healing, PDGF is released by platelets and macrophages. It acts as a chemoattractant, helping to recruit other immune cells to the site of injury. PDGF also stimulates the proliferation of fibroblasts, which are responsible for producing collagen and extracellular matrix. This results in the formation of granulation tissue, which is essential for wound closure and tissue repair. In the final remodeling phase, PDGF continues to support the production of collagen and the restructuring of the tissue, contributing to the restoration of tissue strength and function. Overall, PDGF is a key growth factor in wound healing that helps coordinate the various cellular and molecular processes necessary for the restoration of damaged tissue [27,65].

### 2.8. Effect of F. trijuja Extract Treatment on the Protein Expression Levels of MMP2 and TIMP2 in Wounded Rats

**Figure 7:**
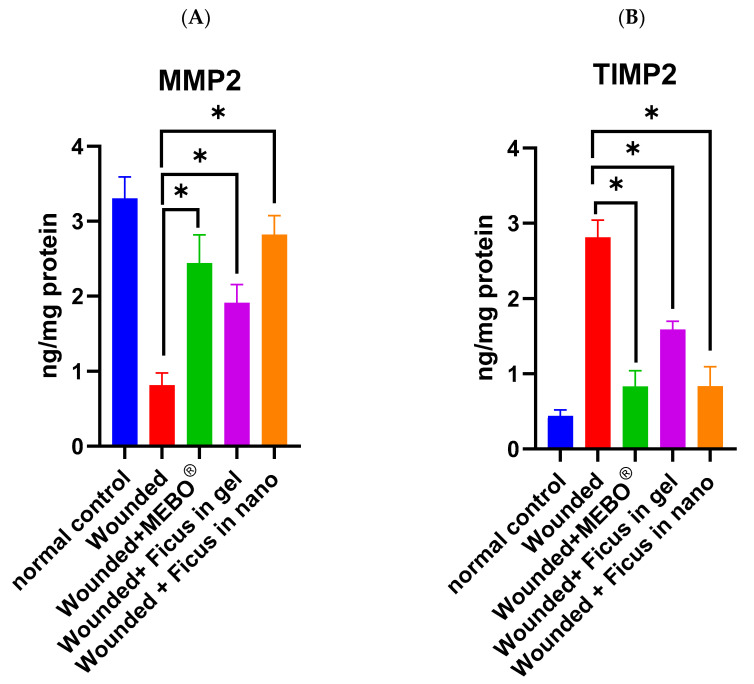
Effect of *F. trijuja* extract treatment on MMP-2 (**A**) and TIMP2 (**B**) protein levels in wounded rats. A total of n = 5 per group was used for all experimental groups to ensure statistical power and reproducibility. Values are expressed as Mean ± SD (n = 5) and analyzed by one-way ANOVA followed by Tukey’s post hoc multiple comparison test. * *p* < 0.05 as compared to the wounded positive control group.

TIMP-2 and MMP-2 are part of a delicate balance responsible for ECM remodeling during the wound healing stage. While MMPs are responsible for breaking down and removing damaged ECM during wound healing, aiding in tissue repair, and facilitating cell migration, TIMP-2 counteracts MMP-2’s excessive activity, preventing aberrant tissue remodeling. The dynamic interaction between these molecules ensures precise tissue repair [28]. In Figure 7A,B, we observed the protein expression levels of MMP2 and TIMP2. The analysis of MMP2 and TIMP2 expression in wounded samples 21 days post-injury reveals noteworthy findings. Wounds treated with *F. trijuja* extract, in both gel and nano-formulation, as well as with MEBO^®^, exhibited a substantial upregulation of MMP2 and a downregulation of TIMP2 compared to untreated wounds (*p* < 0.05).

Remarkably, wounds treated with *F. trijuja* extract in the nano-formulation displayed a similar increase in MMP2 expression and a decrease in TIMP2 expression when compared to the group treated with MEBO^®^. Furthermore, the expression of MMP2 and TIMP2 in wounds treated with *F. trijuja* extract significantly differed between the group treated with MEBO^®^ and the one treated with nano-formulated *F. trijuja* extract, when compared to the gel-formulated *F. trijuja* extract. This evidence supports the conclusion that nano-formulations demonstrate significantly higher efficacy in wound treatment compared to gel-formulations.

### 2.9. Immunohistochemical Staining of Wounded Tissue Using the CD86 Marker

**Figure 8:**
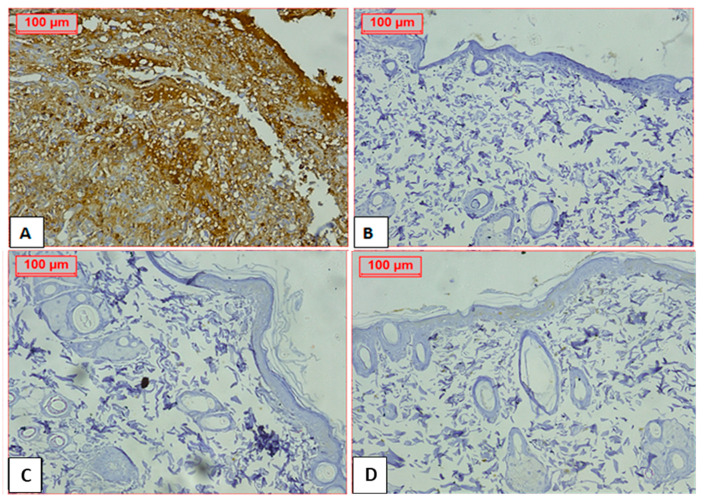
Photomicrographs of CD86-stained histological sections representative of the healing wound area. (**A**): Shows skin wound with strongly positive cells. All other treated groups show negative expression of CD86. (**B**): MEBO^®^ treated group. (**C**): *F. trijuja* extract (in gel formulation) treated group. (**D**): *F. trijuja* extract (in nanoformulation) treated group. (Magnification, 200×).

Macrophages that are traditionally activated, known as “M” macrophages (CD86+), exhibit a pro-inflammatory phenotype. These macrophages release cytokines such as IL-1β and TNFα. They play a crucial role in eliminating pathogens, releasing inflammatory cytokines, and fostering a Th1-type immune response [66,67]. In the context of wound healing, these macrophages are involved in various stages of wound healing, including inflammation, tissue repair, and regeneration [68]. Figure 8 illustrates the immunohistochemical staining of skin tissue of wounded rats on day 21 post-injury using CD86. The MEBO^®^ treated group, *Ficus* (in gel formulation) treated group, and *Ficus* (in nanoformulation) treated group were negative to this marker when compared to the untreated control. These findings provide evidence supporting the wound-healing properties of the *F. trijuja* extract.

## 3. Materials and Methods

### 3.1. Plant Material

Fresh leaves of *F. trijuja* were collected from El-Orman Garden in Giza, Egypt (30°01′45″ N, 31°12′47″ E), in May 2022. Trease Labib, a taxonomy specialist at El-Orman Botanical Garden, authenticated the leaves. A voucher specimen (Code: PHG-P-FT-477) is stored in the herbarium of the Pharmacognosy Department, Faculty of Pharmacy, Ain Shams University, Cairo, Egypt.

### 3.2. Preparation of the Plant Extract

Three kilograms of air-dried *F. trijuja* leaves were macerated in 3 L of 80% methanol and repeated three times at room temperature until fully extracted. The filtrate was concentrated through evaporation under reduced pressure (45 °C) using Rotavap to yield a sticky dried residue, which was further subjected to lyophilization to remove any trace of solvent and yielded 25 g of powdered extract.

### 3.3. LC-HRESIMS Metabolomic Profiling

The 80% methanol extract of *F. trijuja* leaves (1 mg/mL) conducted metabolic examination via LC-HR-ESI-MS following the previously established protocol [69]. An Acquity Ultra Performance Liquid Chromatography (UPLC) system was utilized in conjunction with a Synapt G2 HDMS quadrupole time-of-flight hybrid mass spectrometer (Waters, Milford, MA, USA). Chromatographic separation was performed using a BEH C18 column (2.1 × 100 mm, 1.7 µm particle size; Waters, Milford, USA) equipped with a guard column (2.1 × 5 mm, 1.7 µm particle size). A linear binary solvent gradient from 0% to 100% eluent B was applied over 6 min at a flow rate of 0.3 ml/min, utilizing 0.1% formic acid in water (*v*/*v*) as solvent A and acetonitrile as solvent B. The injection volume was 2 µL, and the column temperature was 40 °C. The total analysis duration for each sample was 20 min. High-resolution mass spectrometry was performed in both positive and negative ESI ionization modes, utilizing a spray voltage of 4.5 kV, a capillary temperature of 320 °C, and a mass range of m/z 150–1500. MZmine 2.20 was used to process the MS dataset and extract data according to the defined parameters. Mass ion peaks have been identified by chromatogram builder and deconvolution. A local minimum search method and isotopic peak grouper were used to differentiate isotopes. A gap-filling peak finder showed missing peaks. Complex and adduct searches were conducted. Following data processing, molecular formula prediction and peak identification were performed. Dereplicated positive and negative ionization mode data from the extract were compared to the DNP (Dictionary of Natural Products).

### 3.4. Preparation of F. trijuja Extract Lipid Nanocapsules (LNCs)

The phase inversion method was trailed to prepare LNCs; then, the system was suddenly cooled using cold water at 0 °C [70]. For the preparation of herbal extract-loaded eucalyptus oil-based LNCs with a final volume 5 mL, 100 mg of the extract was dissolved in 250 mg propylene glycol and mixed with the oil phase before proceeding as follows. Briefly, weighed amounts of Kolliphor^®^ HS15, phospholipon^®^90G, eucalyptus oil, NaCl, and deionized water (Table 3) were mixed together using a heated magnetic stirrer. Heating continued until a milky appearance of the mixture was observed (above PIT; 85 °C), which indicates the formation of W/O emulsion. Then, the mixture was cooled down till the formation of O/W emulsion. Three successive heating–cooling cycles were repeated in the same previous manner. Sudden cooling was made using an equal volume of cold water (0 °C) at the start of the O/W phase in the third cycle. The prepared LNCs were stirred for 10 min for the purpose of particle homogenization.

### 3.5. Preparation of Topical F. trijuja Extract Hydrogel and F. trijuja Extract-Loaded LNC Hydrogels

An equivalent amount of 5% *w*/*w* of carbopol 940 was stirred overnight in distilled water with 0.01% *w*/*v* benzalkonium chloride as a preservative. Then, drops of triethanolamine were added while continuously mixing until a transparent gel was obtained (pH was kept at 7). For the preparation of 1% *F. trijuja* LNCs hydrogel, equal amounts of both the extract-loaded LNCs and the hydrogel were mixed.

For the *F. trijuja* extract hydrogel, 2% *w*/*w* carbopol 940 gel was prepared as described above with 5% propylene glycol added to the gel solution. Then, 1 g of *F. trijuja* extract was added to 90 g of gel and mixed till homogeneity.

### 3.6. Characterization of the Prepared LNCs

#### 3.6.1. Measurement of Particle Size and the Polydispersity Index (PDI)

Photon correlation spectroscopy was used to analyze the particle size and distribution, expressing the LNCs’ average volume diameters and polydispersity index. LNCs were diluted with deionized water before measurement at 25 °C ± 0.5, using a Malvern Zetasizer. All measurements were performed in triplicate.

#### 3.6.2. Determination of Zeta Potential

A laser Doppler Anemometer coupled with the Zetasizer Nano (Zetasizer Nano ZS, Malvern Instruments, Malvern, UK) was used to determine the zeta potential of the prepared LNCs according to electrophoretic light scattering technology. Measurements were carried out in triplicate, at 25 °C ± 0.5.

### 3.7. In Vivo Wound Healing Study

#### 3.7.1. Animals

The animal protocol was approved by the Ethical Committee for Medical Research, National Research Centre, Egypt (No. 01421023). Thirty-six adult Wister rats (150–200 g) from the National Research Centre, Egypt, were housed under pathogen-free conditions with food and water ad libitum. After two weeks of adaptation, the experiments began.

#### 3.7.2. Surgery for the Induction of Skin Injury

**Figure 9:**
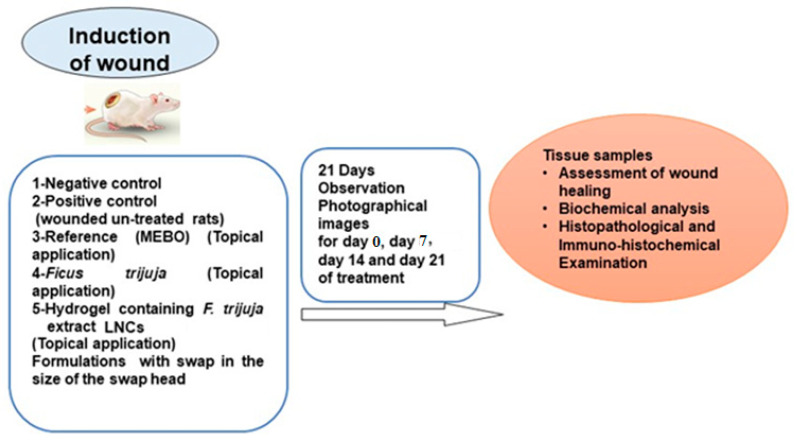
Workflow for the study design.

Rats were anesthetized using ketamine (50 mg/kg, I.M.) and xylazine (5 mg/kg, I.M.). A 15 mm full-thickness skin injury was created on the back [71]. The day of surgery was designated as day zero. Twenty-five rats were assigned to five groups (n = 5): negative control (no wound), positive control (wounded, no treatment), reference (wounded, MEBO^®^ ointment), and two groups treated with different *F. trijuja* topical preparations (Figure 9).

#### 3.7.3. Morphometric and Histopathological Evaluation

Healing was assessed through macro and microscopic observations of the lesions, photographed on days 0, 7, 14, and 21. Specifically, the images were captured with the camera placed approximately 20 cm from the wound site. This distance was maintained consistently across all time points to minimize variability in the image scale. Additionally, we included a scale bar in the images, with a length of 1 cm, to accurately represent the size of the wounds. This will enable more reliable measurements and facilitate a clear comparison of wound healing across groups and time points.

The wound contraction rate was calculated as a percentage reduction in wound size every other day from each rat wound until wound closure. The reduction in the wound size was periodically monitored using transparency paper and a marker. The wound area was assessed graphically to measure the percentage of wound closure, which shows the formation of new epithelial tissue to cover the wound. Wound contraction was expressed as a reduction in the percentage of the original wound size on days 0, 7, 14, and 21. The relative reduction in wound area will be calculated by employing the following equation:

Relative reduction in wound area (%) = ((A_o_ − A_t_))/A_o_ × 100

where, A_o_ and A_t_ are the wound area at zero time and time (t), respectively [72].

### 3.8. Preparation of Tissue Homogenate

Before tissue homogenization, animals were euthanized, and their tissues were meticulously cleaned and rinsed with an ice-cold solution. Afterward, the tissues were gently patted dry using filter paper and weighed on an analytical balance. A 10% homogenate was then prepared in a 0.05 M phosphate buffer (pH 7) utilizing a polytron homogenizer at 4 °C. Subsequently, the homogenate underwent centrifugation at 10,000 rpm for 20 min to eliminate cell debris, unbroken cells, nuclei, erythrocytes, and mitochondria. The resulting supernatant, referred to as the cytoplasmic extract, was employed for the assessment of MMP2, TIMP2, and IL-1β, following the manufacturer’s instructions.

### 3.9. Determination of Tissue Protein

The total tissue protein content was measured using the Bradford method, following the manufacturer’s protocol [73].

### 3.10. Determination of TIMP-2 Protein Levels

TIMP-2 protein levels were determined using TIMP-2 (Rat) ELISA Kit (Biovision, San Jose, CA, USA) according to manufacturer procedures. Briefly, the micro-ELISA plate provided in this kit had been pre-coated with an antibody specific to Rat TIMP-2. Standards or samples were added to the micro-ELISA plate wells that bind to the specific antibody. Then, a biotinylated detection antibody specific to Rat TIMP-2 and Avidin-Horseradish Peroxidase (HRP) conjugate was added successively to each microplate well and incubated. The wells were then washed, a TMB substrate solution was added to the wells, and a blue color developed in proportion to the amount of TIMP-2 bound. The enzyme–substrate reaction was terminated by adding a stop solution and the color turned yellow. The optical density (OD) was measured spectrophotometrically at a wavelength of 450 nm using a microplate reader (Stat Fax 2200, Awareness Technologies, Miami, FL, USA). The concentration of Rat TIMP-2 in the samples can be calculated by comparing the OD of the samples to the standard curve.

### 3.11. Determination of MMP-2 Protein Levels

MMP-2 levels were measured using BioVision’s MMP-2 ELISA kit (Biovision, San Jose, CA, USA), which is based on the Sandwich-ELISA principle according to manufacturer procedures. Briefly, the micro-ELISA plate provided was pre-coated with an antibody specific to rat MMP-2. On the addition of standards or samples to the micro-ELISA plate, they reacted with a biotinylated detection antibody specific for rat MMP-2 and HRP conjugate, which gave a blue color on the addition of substrate. The enzyme–substrate reaction was terminated by adding a stop solution to turn the reaction yellow. The optical density (OD) was determined spectrophotometrically at a wavelength of 450 nm using a microplate reader (Stat Fax 2200, Awareness Technologies, Miami, FL, USA). The concentration of Rat MMP-2 in the samples was calculated by comparing the OD of the samples to the standard curve.

### 3.12. Determination of IL1β Protein Levels

Il-1β protein levels were assessed using an ELISA Kit designed for interleukin 1Beta (IL1β) (manufactured by Cloud Clone Corp., Katy, TX, USA), following the recommended manufacturer’s protocols. Specifically, each well on the plate received an addition of diluted standard, blank, and samples, followed by an incubation period of 1 h at 37 °C. After this incubation, the liquid content was aspirated from each well without any washing steps. Subsequently, Detection Reagent A was introduced into each well and underwent another incubation of 1 h at 37 °C. Following this incubation, the plate underwent three washing processes. After the final wash, Detection Reagent B was introduced to each well and was allowed to incubate for 30 min at 37 °C. This was followed by a washing sequence, which was repeated a total of 5 times as previously described. Substrate Solution was then added to each well, inducing a blue color change, and incubated for 10–20 min at 37 °C. Ultimately, stop solution was dispensed into each well, leading to a noticeable yellow color transformation. A subsequent absorbance measurement of 450 nm was carried out using a microplate reader (Stat Fax 2200, Awareness Technologies, Miami, FL, USA). The corresponding concentration of IL-1β was subsequently calculated based on the generated standard curve.

### 3.13. RNA Extraction

The total RNA was extracted from homogenized tissues using the Direct-zol RNA Miniprep Plus kit (ZYMO research, Irvine, CA, USA) in accordance with the manufacturer’s guidelines. Subsequently, both the quantity and quality of the RNA were evaluated using a Beckman dual spectrophotometer (Beckman Coulter, Brea, CA, USA).

### 3.14. Real-Time PCR

The SuperScript IV One-Step RT-PCR kit (Cat#12594100, Thermo Fisher Scientific, Waltham, MA, USA) was employed to perform reverse transcription on the isolated RNA, followed by PCR amplification. A 96-well plate StepOne instrument (Applied Biosystem, Waltham, MA USA) was utilized, and the thermal profile consisted of an initial 10-min reverse transcription step at 45 °C, followed by a 2-min step at 98 °C for reverse transcription inactivation and initial denaturation, succeeded by 40 cycles of amplification, each comprising 10 s at 98 °C, 10 s at 55 °C, and 30 s at 72 °C.

Following the completion of the RT-PCR, the data were represented in terms of Cycle threshold (Ct) values for both the target genes and the housekeeping gene. To account for variations in the expression of PDGF, VEGF, and TNFα genes, normalization was carried out by referencing the mean critical threshold (Ct) expression value of the GAPDH housekeeping gene, utilizing the ΔΔCt method. The relative quantity of each target gene is quantified according to the calculation of the 2^−∆∆Ct^ method. The primer sequences are represented in (Table 4).

### 3.15. Immunohistochemical Staining

One section from the tissue block of each group was mounted on positively charged glass slides for CD86 staining. Immunostaining was performed using a sensitive avidin-biotin immunoperoxidase technique (Histofine SAB-PO kit, Nichirei, Tokyo, Japan), with no benzidine (DAB) as a chromogenic substrate. The sections were washed with phosphate-buffered saline (PBS) and pre-blocked with rabbit serum for 10 min at room temperature, then incubated with the primary mAb overnight at 4 °C. Sections were then consecutively incubated with biotinylated goat anti-mouse immunoglobulin for 10 min and with peroxidase-conjugated streptavidin for 5 min.

### 3.16. Statistical Analysis

A total of n = 5 per group was used for all experimental groups to ensure statistical power and reproducibility. The results are presented as mean ± standard deviation (SD). A one-way ANOVA followed by Tukey’s/Bonferroni multiple comparisons test was used to assess significance (*p* < 0.05). Analyses were performed using GraphPad Prism software (https://www.graphpad.com/, GraphPad, San Diego, CA, USA).

## 4. Conclusions

This study presents the phytochemical composition and the wound healing abilities of *F. trijuja* leaf extract and its loaded nanocapsules. LC-HRESIMS analysis of *F. trijuja* leaf extract revealed 24 secondary metabolites belonging to different classes of flavonoids, terpenoids, and megastigmane derivatives. Notable for its rich variety of secondary metabolites, the *Ficus* species has been demonstrated to possess antioxidant, anti-inflammatory, antimicrobial, antidiabetic, and cytotoxic properties. The wound healing efficacy of various *Ficus* extracts has been supported by research, including evidence of enhanced wound contraction and strength and shortened epithelization periods. Within the context of herbal extract utilization in wound management, this study provided evidence of the effect of *F. trijuja* on the four phases of wound healing, from hemostasis to maturation, by determining the expression levels of potential wound healing markers as well as staining as in the immunohistochemical study. Additionally, the recent advancements in delivery mechanisms through lipid nanocapsules (LNCs) improved the wound healing capability of the *F. trijuja* extract to that similar to the MEBO^®^ treated group emphasizing the role of formulations and their implications in the development of targeted treatments. Understanding these wound-healing markers at a molecular level provides a foundation for developing targeted therapies to enhance wound healing and mitigate complications in clinical settings.

We acknowledge that lipid carriers may enhance wound healing through mechanisms such as improved skin hydration, barrier repair, or modulation of inflammatory responses. Similarly, gel bases can contribute to wound healing by maintaining an optimal moisture balance in the wound bed, which is essential for tissue regeneration. While the hydration effect of the lipid carriers or gel base may positively influence wound moisturization and re-epithelialization, it is unlikely to significantly affect the anti-inflammatory and biochemical parameters evaluated in the skin, which are specifically modulated by the bioactive extract.

Further studies are thereby recommended for evaluating the effects of the lipid carriers and gel base alone, to rule out their contributions or identify potential synergistic effects when combined with the extract.

## Figures and Tables

**Table 1:** Dereplicated metabolites from LC-HRESIMS analysis of the methanol extract of *Ficus trijuja*.

No.	RT (min.)	*m*/*z*	Ionization Mode	Accurate Mass	Molecular Formula	Metabolite Name	Chemical Class	Reference
1	1.7678892	379.08275	N	380.09002	C_21_H_16_O_7_	Gancaonin F; 9-Hydroxy	Prenylcoumarin	[39]
2	1.7993858	193.07054	P	192.06326	C_7_H_12_O_6_	Quinic acid	Cyclohexanecarboxylic acid	[40]
3	1.9179893	335.09294	N	336.10022	C_20_H_16_O_5_	Carpachromene	Flavonoids	[41]
4	4.674109778	166.08615	P	165.07887	C_9_H_11_NO_2_	Phenylalanine	Amino acid	[42]
5	5.007641778	169.01432	N	170.0216	C_7_H_6_O_5_	Gallic acid	Phenolic acid	[43]
6	8.1189884	327.10715	P	326.09988	C_15_H_18_O_8_	Foveospirolide	Sesquiterpenoid	[44]
7	8.3530242	389.1439	P	388.13662	C_17_H_24_O_10_	*Ficus*carpanoside B	Phenylpropanoid	[51]
8	8.6699946	225.1484	P	224.14112	C_13_H_20_O_3_	9,10-Dihydroxy-4,7- megastigmadien-3- one	Megastigmane	[38]
9	10.403668	507.27947	P	506.2722	C_24_H_42_O_11_	Ficalloside	Megastigmane glycoside	[45]
10	10.471478	539.21185	P	538.20458	C_26_H_34_O_12_	3,3′,4,4′,5,5′,7-Heptahydroxyflavan; 3′,4′,5,5′,7-Penta-Me ether, 3-O-α-L-rhamnopyranoside	Flavonoid	[9]
11	11.035368	595.16502	P	594.15774	C_27_H_30_O_15_	Sorbifolin-6-O-[*α*-L-arabinopyranosyl-(1→2)-*β*-D-glucopyranoside]	Flavonoid	[34]
12	11.035501	611.15993	P	610.15265	C_27_H_30_O_16_	Rutin	Flavonoid	[35]
13	11.093782	511.18053	P	510.17326	C_24_H_30_O_12_	5, 7, 3 Trimethoxy leucodelphinidin 3-O-*α*-L-Rhamnoside	Leucoanthocyanidin	[36]
14	11.38093	197.11712	P	196.10985	C_11_H_16_O_3_	*Ficus*ic acid	Monoterpenoid	[37]
15	12.467357	399.12809	P	398.12082	C_18_H_22_O_10_	6-Carboxyethyl-7- methoxyl-5-hydroxy-benzofuran 5-O-*β*-D-glucopyranoside	Benzofuran Glucoside	[52]
16	19.130958	469.33085	P	468.32357	C_30_H_44_O_4_	Foveolide B	Sesquiterpenoid dimer	[44]
17	20.171168	473.36217	P	472.35489	C_30_H_48_O_4_	29,30-Dinor-3*β*-acetoxy-18,19-dioxo-18,19-secolupane	Triterpenoid	[46]
18	20.591852	457.36732	P	456.36004	C_30_H_48_O_3_	19,20-secoursane-3,19,20-trione	Triterpenoid	[47]
19	21.175209	457.36733	P	456.36005	C_30_H_48_O_3_	3*α*-Hydroxyisohop-22(29)-en-24-oic acid	Triterpenoid	[50]
20	23.35141	279.15897	P	278.15169	C_16_H_22_O_4_	(+)-(*R*)-de-O-Methyllasiodiplodin	Benzoic acid lactones	[53]
21	25.980935	459.38296	P	458.37569	C_30_H_50_O_3_	3*β*-hydroxy-11*α*-hydroperoxy-12-ursene	Triterpenoid	[48]
22	26.121905	427.39316	P	426.38589	C_30_H_50_O	Rhoiptelenol	Triterpenoid	[50]
23	27.17658	443.3881	P	442.38083	C_30_H_50_O_2_	20*α*,21*α*-Epoxytaraxastan-3*β*-ol	Triterpenoid	[49]
24	27.364269	441.37248	P	440.3652	C_30_H_48_O_2_	29(20→19) Abeolupane-3,20-dione	Triterpenoid	[47]

RT (min.): retention time per mint, *m*/*z*: mass to charge.

**Table 2:** Evaluation of wound healing. A total of n = 5 per group was used for all experimental groups to ensure statistical power and reproducibility. Values are presented as Mean ± SD. *: significantly different from the corresponding positive control (wounded) at *p* < 0.05 using repeated One-way ANOVA followed by Bonferroni as a post hoc test.

	Control Positive	MEBO^®^	*F. trijuja*	*F. trijuja* (LNCs)
Day 0	6.18 ± 0.42	34.58 ± 1.66 *	29.12 ± 1.70 *	25.89 ± 2.29 *
Day 7	12.35 ± 0.84	69.16 ± 3.31 *	58.25 ± 3.41 *	51.78 ± 4.58 *
Day 14	44.90 ± 2.68	89.11 ± 1.23 *	71.44 ± 2.59 *	94.77 ± 0.65 *
Day 21	63.22 ± 2.39	95.77 ± 0.46 *	96.73 ± 0.59 *	98.28 ± 0.06 *

**Table 3:** Detailed composition of loaded eucalyptus oil-based LNCs loaded with herbal extract.

Constituents	Percentage (%*w*/*w*)
Herbal extract	2%
Propylene glycol	5%
Eucalyptus oil	15%
Kolliphor^®^ HS15	15%
Phospholipon^®^90G	1%
Sodium Chloride	1%
Deionized water	61%

**Table 4:** Primer sequences used for the RT-PCR.

Gene Symbol	Forward	Reverse	Gene Bank Accession
PDGF	TGAGTGGCGAGCACTGTC	TCTGGATTTCCCAGCTGT	AB003156.1
VEGF	GGCTCTGAAACCATGAACTTTCT	GCAGTAGCTGCGCTGGTAGAC	NM_001287114.1
TNFα	TGCCTCAGCCTCTTCTCATT	GAGCCCATTTGGGAACTTCT	NM_012675.3
GAPDH	GTGCCAACCCCAAACGTATC	CTGCTTTCACAGCCTCCTTGA	NM_023964.1

## Data Availability

All data generated or analyzed during this study are included in this article and its Supplementary Information Files.

## Supplementary Materials

The following supporting information can be downloaded at https://www.mdpi.com/article/10.3390/ijms26030928/s1.

## Abbreviations

Lipid nanocapsules	(LNCs)
Medium chain triglycerides	(MCT)
Polyethylene glycol	(PEG)
Tumor Necrosis Factor-alpha	(TNF-α)
Interleukin 1Beta	(IL1β)
Vascular Endothelial Growth Factor	(VEGF)
Platelet-Derived Endothelial Growth Factor	(PDGF)
Tissue Inhibitor of Metalloproteinases 2	(TIMP2)
Matrix Metalloproteinase 2	(MMP2)
Optical density	(OD)
Polydispersity index	(PDI)
Horseradish Peroxidase	(HRP)
Cycle threshold	(Ct)
Phosphate-buffered saline	(PBS)
Relative quantitation	(RQ)
Standard deviation	(SD)
Analysis of variance	(ANOVA)
