# Supplementary material for: Mechanistic Wound Healing of Ficus trijuja Leaf Extract and Its Lipid Nanocapsule Supported by Metabolomic Profiling and In Vivo Studies
Source: Int J Mol Sci. 2025 Jan 23;26(3):928. doi: 10.3390/ijms26030928 (PMC11817089; doi:10.3390/ijms26030928)
Supplement: Supplementary file 1 [file ijms-26-00928-s001.zip › ijms-3357334-supplementary.pdf]

Supplementary material

Mechanistic Wound Healing of *Ficus trijuja* Leaves Extract and Its Lipid Nanocapsule Supported by Metabolomic Profiling and *In-Vivo* Studies

Ingy M. Hashad [1], Shaza H. Aly [2], Dalia O. Saleh [3], Nesma M. E. Abo El-Nasr [3], Marwa E. Shabana [3], Fatma Sa'eed El-Tokhy [4], Heba A.S. El-Nashar [5], Usama R. Abdelmohsen [6,7], Nada M. Mostafa [5*] and Ahmed M. Mostafa [8*]

[1] Department of Biochemistry, Faculty of Pharmacy and Biotechnology, German University in Cairo, Cairo 11835, Egypt.
[2] Department of Pharmacognosy, Faculty of Pharmacy, Badr University in Cairo (BUC), Cairo 11829, Egypt.
[3] Department of Pharmacology, National Research Centre (NRC), Cairo 12622, Egypt.
[4] Department of Pharmaceutics and Pharmaceutical Technology, Faculty of Pharmacy, Badr University in Cairo (BUC), Cairo 11829, Egypt.
[5] Department of Pharmacognosy, Faculty of Pharmacy, Ain Shams University, Cairo 11566, Egypt.
[6] Department of Pharmacognosy, Faculty of Pharmacy, Deraya University, New Minia 61111, Egypt.
[7] Department of Pharmacognosy, Faculty of Pharmacy, Minia University, Minia 61519, Egypt
[8] Department of Biochemistry, Faculty of Pharmacy, Ain Shams University, Cairo 11566, Egypt.
* Correspondence: authors: nadamostafa@pharma.asu.edu.eg (N.M.M.); ahmedmostafa@pharma.asu.edu.eg (A.M.M.)

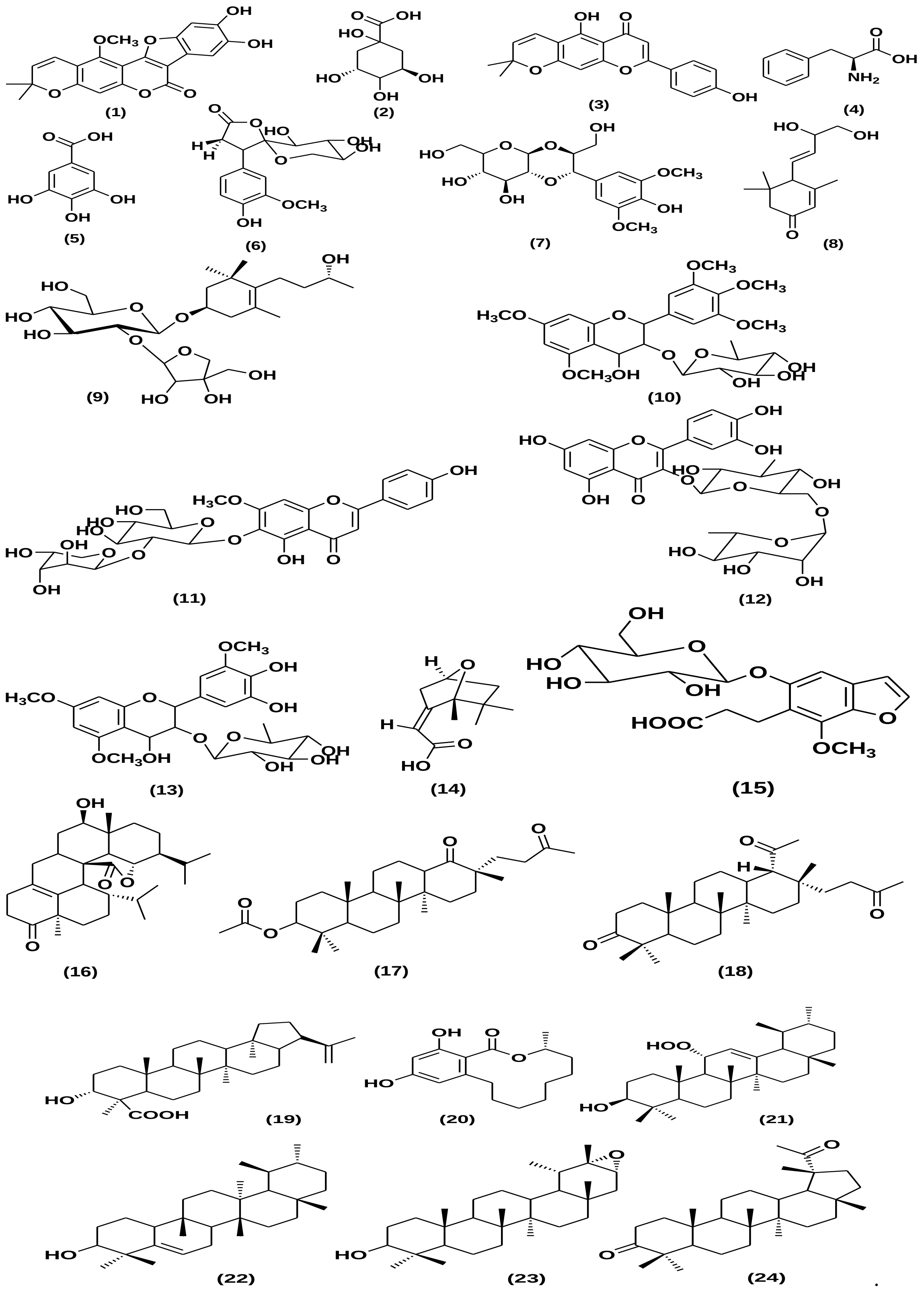

Figure S1. Dereplicated metabolites from LC-HRESIMS analysis of methanol extract of *Ficus trijuja.*

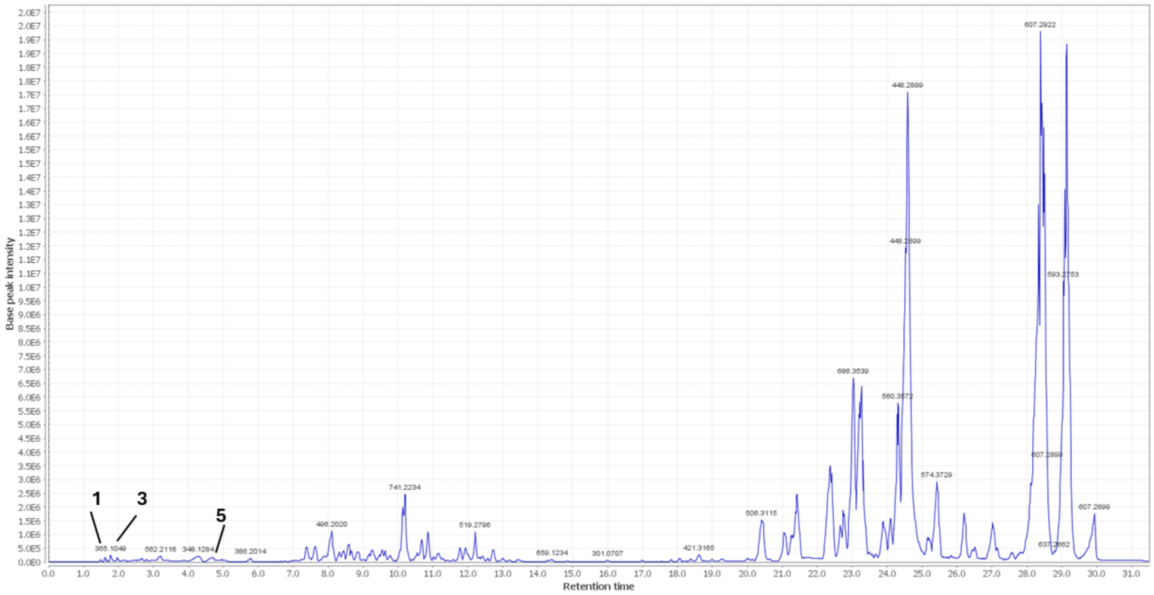

Figure S2. LC-HRESIMS chromatogram of the dereplicated metabolites of methanol extract of *Ficus trijuja* (Negative).

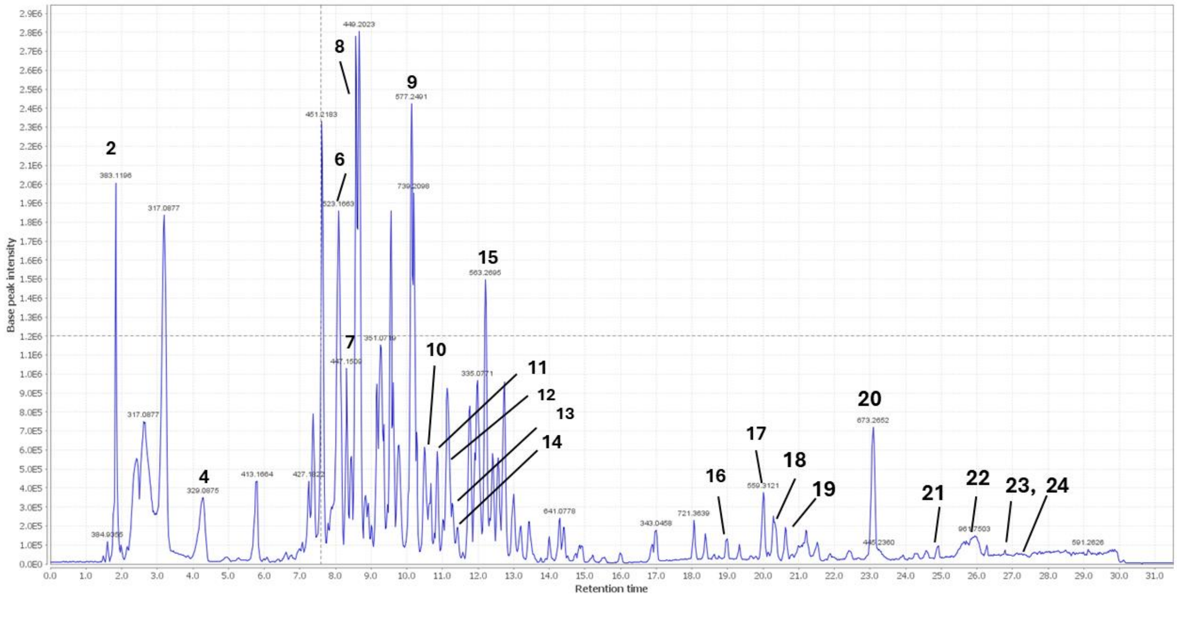

Figure S3. LC-HRESIMS chromatogram of the dereplicated metabolites of methanol extract of *Ficus trijuja* (Positive).
